# Epidemiology of Orthopaedic Trauma Admissions From the Emergency Department of a Major Trauma Centre in Rural North India

**DOI:** 10.7759/cureus.109049

**Published:** 2026-05-17

**Authors:** Jasveer Singh, Dinesh Kumar, Ankit Mittal, Santosh Kumar Singh, Sohan Yadav, Ajay K Rajput, Pradeep K Gupta, Ajay K Saroj

**Affiliations:** 1 Orthopaedics, Uttar Pradesh University of Medical Sciences (UPUMS) Saifai, Saifai, IND; 2 Orthopaedic Surgery, Uttar Pradesh University of Medical Sciences (UPUMS) Saifai, Saifai, IND; 3 Orthopaedics and Traumatology, Maa Vindhyawasini Autonomous State Medical College, Mirzapur, IND; 4 Orthopaedics and Trauma, Uttar Pradesh University of Medical Sciences (UPUMS) Saifai, Saifai, IND

**Keywords:** emergency department, injury patterns, orthopaedic injuries, orthopaedic patients, road traffic accidents

## Abstract

Orthopaedic trauma remains a major public health problem, particularly in rural India, where preventive strategies and trauma-care resources are often limited. This single‑centre descriptive observational study aimed to describe the epidemiological profile of orthopaedic trauma patients admitted to a major trauma centre in rural North India, with emphasis on age, sex, mechanism of injury, and pattern of skeletal involvement.

This observational study included 2,340 consecutive patients of all ages admitted under orthopaedics with acute traumatic injuries after initial presentation to the emergency department between December 2022 and December 2024. Repeat admissions for the same injury episode were excluded, and polytrauma patients were included if they had at least one orthopaedic injury. Data were collected prospectively from patient interviews and direct observation, and supplemented by the hospital medical record system.

Descriptive statistics were used to analyse the demographic characteristics, mechanisms of injury, injury types, and anatomical fracture sites; age‑group differences in selected injury categories were analysed using chi‑square tests.

Out of 2,340 patients, the study population consisted of 1,760 (75.21%) male and 580 (24.79%) female patients. The most common age group was 20-29 years (480; 20.51%), followed by 30-39 years (419; 17.91%) and 10-19 years (390; 16.67%). Road traffic accidents were the leading mechanism of injury (1,242; 53.08%), followed by slip-and-fall on the ground (394; 16.84%) and falls from height (385; 16.45%). Other mechanisms included mechanical injury (98; 4.19%), hit by animal (88; 3.76%), assault (67; 2.86%), fall of heavy object (32; 1.37%), railway injury (18; 0.77%), and sports injury (16; 0.68%).

Fractures were the most common injury type (2,182; 93.25%), followed by dislocations (80; 3.42%) and soft tissue injuries (78; 3.33%). Limb amputation/crush injury was recorded in 80 patients (3.42%). Single-bone involvement was seen in 2,006 patients (85.73%), while 176 (7.52%) had more than one fractured bone. Open fractures were 445 (19.02%) cases, and closed fractures were 1,737 (74.23%).

The commonly involved anatomic sites included femur, distal radius, both-bone leg, ankle region, and foot. The number of recorded fracture sites exceeded the number of fracture patients because some patients had bilateral or multi-site injuries. In age-wise analysis, dislocations were most frequent in the 20-39-year group, while limb amputation/crush injuries and open fractures were most common in the 40-59-year group. There was no significant overall association between age group and selected injury categories.

Most patients of orthopaedic trauma in rural North India are young adult males, most commonly injured in road traffic accidents. Fractures remain the leading injury pattern, with the femur and distal radius among the most commonly involved sites. Preventive strategies should focus on road safety, occupational protection, and strengthening trauma-care services.

## Introduction

Trauma remains one of the leading causes of morbidity and mortality worldwide and continues to be a major public health problem in both developed and developing countries [1-4]. Road traffic injuries are expected to remain a major contributor to this burden in the coming decades, particularly in low- and middle-income countries where rapid urbanization, weak enforcement of safety measures, unsafe workplaces, and limited access to timely trauma care worsen outcomes [1,4,5]. Recent reviews of trauma care in India also emphasize that, although progress has been made, the system remains fragmented and uneven across regions [1,4,5].

Orthopaedic trauma constitutes a large proportion of emergency department work and is commonly caused by road traffic accidents, falls, sports injuries, and work-related injuries [2,3]. These injuries may lead to pain, disability, loss of ability to earn, prolonged treatment, and considerable socioeconomic burden on patients and families [2,3]. In rural areas these problems are often compounded by delayed transport, limited referral pathways, and reduced access to specialist care [1,4,5].

The concept of golden hour is still relevant in trauma care as early stabilisation and timely transfer to an appropriately equipped centre can reduce morbidity and improve outcome [1,4,5]. However, such care is not always readily available in rural North India, where trauma infrastructure and injury prevention awareness are uneven [1,4,5]. Recent Indian trauma series have also shown that road traffic injuries, young adult males, and two-wheeler-related crashes form a major share of the trauma burden [1-3].

However, most existing Indian trauma series focus on urban level‑I centres or mixed trauma cohorts and provide limited information on detailed anatomical fracture distribution or age‑specific clustering of severe orthopaedic injuries in rural major trauma centres. There is a particular paucity of data from rural North India regarding orthopaedic trauma admissions, where referral patterns and resource constraints may differ substantially from urban settings.

Thus, a good understanding of the epidemiology of orthopaedic trauma is crucial in planning preventive strategies, strengthening trauma systems, and allocating resources more efficiently [1,2,4]. Information regarding age and sex distribution, mechanisms of injury, and skeletal involvement helps identify high‑risk groups and injury patterns, including age‑specific clustering of severe trauma [2,3,6].

This study was designed to evaluate these factors in patients admitted to a major trauma centre in rural North India.

## Materials and methods

This single‑centre prospective descriptive observational study was conducted in the Department of Orthopaedics, Uttar Pradesh University of Medical Sciences (UPUMS) Saifai, Etawah, over a period of two year from December 2022 to December 2024. UPUMS functions as a major trauma referral centre for rural districts in western Uttar Pradesh, with an approximate catchment population of two million, and receives secondary and tertiary referrals from peripheral health centres and smaller hospitals. A total of 2,340 patients with orthopaedic trauma were included after approval from the Institutional Ethics Committee (Ethical clearance number 560/UPUMS/DSW Ethical/ 111/ 2022-23). Patients of all age groups were eligible if they presented with orthopaedic trauma during the study period.

Patients who refused to participate or had chronic orthopaedic illness were excluded. Data collection was prospective. For each eligible patient, a structured proforma was completed at admission based on patient interview, direct clinical assessment and initial investigations and was supplemented by review of the electronic and paper medical records during the hospital stay.

Data were collected by patient interview, medical record review, direct observation, and hospital electronic medical record system. Information on age, sex, injury mechanism, anatomical site, injury type, fracture pattern, and comorbidities was recorded.

Injuries were classified according to anatomical site and type of fracture.

Data were analysed using SPSS version 24 (IBM Corp., Armonk, NY, USA). Data were summarized using descriptive statistics. Continuous variables were summarised as mean and standard deviation, and categorical variables such as sex, age group, injury mechanism, and injury type were expressed as frequencies and percentages. Age‑group differences in the proportions of selected injury categories (dislocations, limb amputation, crush injury and open fractures) were assessed using the chi‑square test of independence. A p‑value < 0.05 was considered statistically significant.

## Results

A total of 2,340 patients with orthopaedic trauma were included in the study. Males accounted for 1,760 cases (75.21%), while females accounted for 580 cases (24.79%). The highest burden was among young adults, especially those aged 20-29 years (480 patients, 20.51%), followed by those aged 30-39 years (419 patients, 17.91%) and those aged 10-19 years (390 patients, 16.67%).

Road traffic accidents were the most common mechanism of injury in 1,242 cases (53.08%). This was followed by slip-and-fall on the ground in 394 patients (16.84%) and falls from height in 385 patients (16.45%). Mechanical injury was recorded in 98 patients (4.19%), hit by animal in 88 (3.76%), assault in 67 (2.86%), fall of heavy object in 32 (1.37%), railway injury in 18 (0.77%), and sports injury in 16 (0.68%) (Table 1).

**Table 1:** Demographic profile of orthopaedic trauma patients (N = 2340). Data are presented as n and %. RTA: road traffic accident

Variable	Category		n	%
Sex	Male		1760	75.21
	Female		580	24.79
Age group (years)	0-9		230	9.83
	10-19		390	16.67
	20-29		480	20.51
	30-39		419	17.91
	40-49		280	11.97
	50-59		230	9.83
	60-69		210	8.97
	>69		101	4.32
Mechanism of injury	RTA	Pedestrian	42	1.79
		Bicycle	90	3.85
		Motorcycle	548	23.42
		Car	370	15.81
		Heavy vehicle	192	8.21
	Slip on ground		394	16.84
	Fall from height		385	16.45
	Mechanical injury		98	4.19
	Hit by animal		88	3.76
	Assault		67	2.86
	Fall of heavy object		32	1.37
	Railway injury		18	0.77
	Sport injury		16	0.68

Fracture was the commonest type of injury involving 2,182 (93.25%) patients. Dislocation was observed in 80 (3.42%) patients, while other injuries such as ligament tear, tendon injury, and soft tissue injury were seen in 78 (3.33%) patients. Limb amputation and crush injury were observed in 80 (3.42%) patients (Table 2).

**Table 2:** Injury characteristics of the patients. Data are presented as n and %.

Injury	Category	n	%
Injury type	Fractures	2182	93.25
	Isolated joint dislocations	80	3.42
	Soft tissue injuries	78	3.33
Limb amputation/crush injury	Yes	80	3.42
Number of fractured bones	1 bone	2006	85.73
	>1 bone	176	7.52
Fracture type	Open fracture	445	19.02
	Closed fracture	1737	74.23

The anatomical distribution of fractures showed involvement of clavicle (112; 5.13%), distal radius (168; 7.70%) and metacarpals and phalanges (96; 4.40%) were common upper‑limb fracture sites, while femoral shaft (195; 8.94%), intertrochanteric (137; 6.28%) and both‑bone leg fractures (218; 9.99%) dominated lower‑limb injuries (Table 3).

**Table 3:** Anatomical distribution of recorded fracture sites among fracture patients (n = 2,182). Data are presented as n and %, calculated with reference to 2,182 fracture patients (N = 2,182 fracture patients; total recorded fracture sites = 2,435).

Bone involved	Specific fracture site	n	%
Isolated clavicle		112	5.13
Isolated scapula		32	1.47
Isolated humerus	Proximal	38	1.74
	Shaft	56	2.57
	Distal	92	4.22
Isolated radius	Head	28	1.28
	Shaft	56	2.57
	Distal radius	168	7.70
Isolated ulna	Olecranon	32	1.47
	Shaft	46	2.11
	Distal	28	1.28
Both bone forearm		99	4.54
Isolated hand	Scaphoid	13	0.60
	Other carpals	9	0.41
	Metacarpals and phalanges	96	4.40
Spine		38	1.74
Pelvic fractures	Pubic rami	71	3.25
	Sacrum	1	0.05
	Acetabulum	18	0.82
	Iliac blade	10	0.46
Isolated femur	Head and neck	85	3.90
	Intertrochanteric	137	6.28
	Subtrochanteric	28	1.28
	Shaft	195	8.94
	Distal femur	88	4.03
Isolated patella		96	4.40
Isolated tibia	Proximal	91	4.17
	Shaft	86	3.94
	Distal	19	0.87
Isolated fibula	Head	18	0.82
	Shaft	42	1.92
Both bone leg		218	9.99
Ankle	Medial malleolus	35	1.60
	Lateral malleolus	28	1.28
	Posterior malleolus	46	2.11
	Tri malleolar	7	0.32
Foot	Calcaneus	35	1.60
	Talus	8	0.37
	Other tarsals	10	0.46
	Metatarsals and phalanges	120	5.50

Pelvic fractures included pubic rami fractures in 71 patients (3.25%), sacral fracture in one (0.05%), acetabular fractures in 18 (0.82%), iliac blade fractures in 10 (0.46%), and coccygeal injury in 0 patients. Femoral injuries were common with head and neck fractures in 85 cases (3.90%), intertrochanteric fractures in 137 (6.28%), subtrochanteric fractures in 28 (1.28%), shaft fractures in 195 (8.94%), and distal femoral fractures in 88 (4.03%). Patellar fractures were recorded in 96 patients (4.40%).

In 91 patients (4.17%) tibial fractures were classified as proximal tibial fractures, in 86 (3.94%) as tibial shaft fractures, and in 19 (0.87%) as distal tibial fractures. Fibular fractures included head fractures in 18 patients (0.82%) and shaft fractures in 42 (1.92%). Both-bone leg fractures were one of the major injury patterns, accounting for 218 cases (9.99%). At the ankle, medial malleolar fractures were seen in 35 cases (1.60%), lateral malleolar fractures in 28 (1.28%), bimalleolar fractures in 46 (2.11%), and trimalleolar fractures in seven (0.32%). Calcaneal fractures were recorded in 35 patients (1.60%), talus fractures in eight (0.37%), other tarsal fractures in 10 (0.46%), and metatarsal and toe fractures in 120 (5.50%). Axial skeleton was also involved. Spinal injuries were noted in 38 patients (1.74%) (Table 3).

Shoulder dislocation was noted in 28 cases (1.20%); elbow dislocation in 12 (0.51%); hip dislocation in 35 (1.50%), and knee dislocation or subluxation in two patients (0.09%). Crush injury resulting in amputation of upper limb was noted in 33 cases (1.41%) and crush injury and amputation of lower limb were noted in four cases (1.17%) (Table 4).

**Table 4:** Distribution of crush injuries/amputations and joint dislocations (N = 2340)

Injury	Location	n	%
Crush injuries and amputation	Upper limb	33	1.41
	Lower limb	4	0.17
Isolated joint dislocation	Shoulder	28	1.20
	Elbow	12	0.51
	Hip	35	1.50
	Knee	2	0.09

Trauma involved a single bone in 2,164 patients (92.48%) and more than one bone in 176 patients (7.52%). Open fractures were seen in 445 patients (19.02%) and closed fractures in 1,895 patients (80.98%). In road traffic accident cases, the commonest vehicles involved were motorcycles in 548 cases (44.12%), followed by cars in 370 cases (29.79%), heavy vehicles in 192 (15.46%), bicycles in 90 (7.25%), and pedestrians in 42 (3.38%).

When age groups were compared, dislocations were most common in the 20-39-year group (39 cases; 4.34%), limb amputation and crush injuries were highest in the 40-59-year group (28 cases; 5.49%), and open fractures were most frequent in the 40-59-year group (155 cases; 30.39%). When age groups were compared, there was no statistically significant difference in the proportion of dislocations across age categories (χ² = 5.66, p = 0.130). In contrast, limb amputation and crush injuries showed a significant age‑related variation, with the highest proportion observed in the 40-59‑year group (χ² = 12.75, p = 0.005). Open fractures also demonstrated a significant association with age, rising progressively and peaking in the 40-59‑year group before declining in older patients (χ² = 16.32, p < 0.001) (Table 5).

**Table 5:** Age-group distribution of selected injury categories. Data are presented as n and % within each age group. Chi‑square test of independence (df = 3) was used to compare proportions across age groups; χ² statistics and p‑values are shown. Statistical significance was defined as p < 0.05.

Injury category	0-19 years (n=620)	20-39 years (n=899)	40-59 years (n=510)	60-79 years (n=311)	χ²	p-value
Dislocations	13 (2.10)	39 (4.34)	18 (3.53)	10 (3.22)	5.66	0.130
Limb amputation and crush injury	10 (1.61)	30 (3.34)	28 (5.49)	12 (3.86)	12.75	0.005
Open fracture	58 (9.35)	192 (21.36)	155 (30.39)	40 (12.86)	16.32	<0.001

## Discussion

The present study showed that orthopaedic trauma in rural North India predominantly affected young adult males and was most commonly caused by road traffic accidents. This pattern is consistent with James et al. [1] and Singh et al. [2], both of whom reported that trauma in India continues to be concentrated in young men and is largely driven by high-energy mechanisms.

The male predominance in this study probably reflects greater exposure of men to road travel, outdoor work, agricultural labour, and other high-risk activities. Similar gender patterns were reported by Joshipura et al. [5] and Rohilla et al. [3], who also observed that orthopaedic trauma remains more common in economically active male populations.

Young adults formed the largest affected age group in the present study. This is clinically important because injuries in this group affect the most economically productive segment of the population. A similar age pattern was seen by Sen et al. [7] and Kandel et al. [8], both of whom reported that major orthopaedic trauma is concentrated in younger patients.

Road traffic accidents were the most common mechanism of injury in this series. This finding is in keeping with James et al. [1] and Singh et al. [2], who identified road traffic injury as a major cause of trauma admissions. In our study, motorcycles were the most common vehicle involved, which is particularly relevant in India, where two-wheeler crashes continue to produce a large burden of severe orthopaedic trauma. This supports the need for better prehospital trauma care, helmet compliance, and stronger road safety enforcement.

Falls were the second most frequent mechanism of injury, especially falls on the ground and falls from height, probably reflecting occupational hazards, environmental risk, and age-related frailty in older patients. Similar observations were made by Mohan et al. [6], who also reported that falls remain an important mechanism of orthopaedic injury. Preventive measures should therefore address both workplace safety and fall prevention in the elderly.

Fractures were the most common injury in this study, which is to be expected in an orthopedic trauma cohort. The high fracture burden is also consistent with Mohan et al. [6] and Candela et al. [9], both of whom showed that fractures form the major share of hospital-based orthopaedic injuries. Lower limb injuries, particularly around the femur, tibia, ankle, and foot, formed a major proportion of the trauma burden in our series. These injuries often require operative treatment, longer rehabilitation, and more hospital resources.

Femoral fractures were one of the most common injuries in the present study, especially shaft and intertrochanteric fractures. High-energy trauma remains the most common cause of femoral injury in younger adults, while proximal femoral fractures are also seen after low-energy falls in older patients. This trend is similar to the findings of Sen et al. [7], who highlighted the role of high velocity trauma in major lower limb and pelvic injuries in India. These findings have important implications for trauma system preparedness, availability of implants, and surgical expertise.

Injuries to the upper limb, including fractures of the distal radius, humerus, forearm, clavicle, and hand, were also common. Such injuries are often the result of falls on an outstretched hand, direct impact, or road traffic trauma. The high frequency of distal radius injuries is supported by Candela et al. [9], who reported a similar fracture pattern in a hospital-based orthopaedic practice. Although upper limb injuries may appear less severe than femoral or pelvic trauma, they can still result in significant loss of function and delayed return to work.

Open fractures, dislocations, amputations, and crush injuries were a smaller but clinically significant component of the injury burden. Open fractures need urgent debridement and fixation because of the high risk of infection. The trauma system review by James et al. [1] and the retrospective trauma analysis by Singh et al. [2] emphasise the importance of timely prehospital care, appropriate referral pathways, and organised trauma services. Dislocations and crush injuries in our study involved young and middle-aged adults, suggestive of exposure to high-energy mechanisms and occupational trauma.

In summary, the results of this study support the need for a comprehensive trauma-prevention strategy in rural North India. Priority areas include enforcement of road safety, helmet and seatbelt use, safer two-wheeler riding, occupational injury prevention, and fall-prevention measures [1,2,4]. Trauma centers must also be strengthened in terms of triage, timely imaging, surgical capacity, blood availability, rehabilitation services, and referral networks, especially given the trauma-system inequalities noted in multicentred studies [1,4,5,10,11]. Without these measures, the burden of orthopaedic trauma will continue to fall disproportionately on young, productive individuals.

This study has several limitations. First, it was conducted at a single rural tertiary care centre, which may limit the generalisability of the findings to other regions or urban settings. Second, the observational design relied on hospital records and patient recall, so some pre‑hospital and mechanism‑related details may be incomplete or subject to reporting bias. Third, we focused primarily on epidemiological patterns and did not evaluate treatment outcomes, functional recovery, or long‑term disability, which are important for assessing the full burden of trauma. Future multicentre studies incorporating outcome measures are needed to build on these findings.

## Conclusions

Severe injuries such as open fractures and limb amputation or crush injuries were particularly frequent in middle‑aged adults, highlighting the need for targeted preventive and trauma‑care strategies for this high‑risk group. Targeted road safety interventions for two‑wheeler users, workplace injury‑prevention measures, and strengthening rural trauma referral pathways may help reduce the burden of orthopaedic trauma in similar settings.
